# Serological Evidence of Flavivirus Exposure and Limited Avian Influenza Exposure in Urban House Martins from Southwestern Spain

**DOI:** 10.3390/ani16060913

**Published:** 2026-03-13

**Authors:** Irene Hernandez-Caballero, Luz García-Longoria, Carlos Mora-Rubio, Sergio Magallanes, João T. Cruz, Alazne Díez-Fernández, Wendy Flores-Saavedra, Alfonso Marzal

**Affiliations:** 1 Department of Anatomy, Cellular Biology and Zoology, University of Extremadura, 06006 Badajoz, Spain; 2 Department of Conservation Biology and Global Change, Doñana Biological Station—Spanish National Research Council (EBD-CSIC), 41092 Seville, Spain; 3 Biomedical Research Networking Center for Epidemiology and Public Health (CIBERESP), 28029 Madrid, Spain; 4 Centre for Interdisciplinary Research in Animal Health (CIISA), Faculty of Veterinary Medicine (FMV-ULisboa), University of Lisbon, 1300-477 Lisbon, Portugal; 5 Associate Laboratory for Animal and Veterinary Sciences (AL4AnimalS), 1300-477 Lisbon, Portugal; 6 Wildlife Research Group, National University of San Martín, Jr. Maynas 1777, Tarapoto 22021, Peru

**Keywords:** animal health, *Delichon urbicum*, diagnostic, epidemiology, One Health, surveillance, zoonotic diseases

## Abstract

Zoonotic diseases account for approximately one billion cases of illness and millions of deaths globally each year. Increasing contact between humans and competent wildlife hosts elevates the risk of zoonotic spillover. Synanthropic bird species are key players in the transmission of zoonotic pathogens, including flaviviruses such as West Nile virus (WNV) and influenza A viruses like Avian Influenza Virus (AIV). Active surveillance of sentinel birds inhabiting urban areas allows for early detection of emerging pathogens before they cause zoonotic outbreaks. Despite nesting in close proximity to humans, the role of the house martin (*Delichon urbicum*) in the circulation of flaviviruses and AIV remains poorly understood. Here, we analyzed the presence of antibodies against flaviviruses and AIV in a colony of house martins from southwestern Spain. In addition, we aimed to detect amplicons of the matrix and nucleoprotein genes of AIV using RT-qPCR. While none of the samples tested positive for AIV by RT-qPCR, we observed an AIV seroprevalence of 2.13% based on non-subtyped ELISA. Notably, this is the first report of AIV-seropositive *D. urbicum* individuals captured in Spain. Moreover, we detected a flavivirus-group seroprevalence of 24.34%, similar to rates reported in the same house martin population between 2018 and 2020, suggesting widespread circulation of flaviviruses within this synanthropic species. These results support the hypothesis that house martins may participate in the transmission of these viruses between wild bird populations and humans in urban environments.

## Simple Summary

Emerging infectious diseases pose a growing threat to human health worldwide, particularly those caused by zoonotic pathogens that can spread from animals to people. Birds living in urban areas can carry these pathogens and serve as early indicators of outbreaks. In this study, we investigated a colony of house martins (*Delichon urbicum*) in southwestern Spain to assess exposure to avian influenza virus and flaviviruses. Although no viral RNA was detected, a small proportion of birds had antibodies against avian influenza, and nearly one-quarter had antibodies against flaviviruses. These findings suggest that house martins may contribute to the transmission of these viruses, highlighting their importance for monitoring emerging zoonotic diseases in urban environments.

## 1. Introduction

It is estimated that globally, approximately one billion cases of illness and millions of deaths occur annually due to zoonoses—infectious diseases caused by pathogens transmitted between animals and humans [1]. While this issue is not new, the last century has witnessed a notable rise in emerging and re-emerging zoonoses, with over 30 new human pathogens identified during the last 30 years, 75% originating from animals [2]. These include both newly appearing diseases and those that previously existed but now are increasing rapidly in incidence or geographic spread, posing growing public health threats [3] like Japanese Encephalitis Virus (JEV), Tick-Borne Encephalitis Virus (TBEV), Usutu Virus (USUV), Zika virus (ZIKAV), Dengue Virus (DENV) or West Nile Virus (WNV) [4]. Wild birds are important to public health because they play crucial roles in the transmission and spread of emerging zoonotic pathogens [5]. For instance, Desvars-Larrive et al. [6], analyzing data from 1975 to 2022 in Austria, identified over 80 bird species carrying zoonotic pathogens. Notably, birds were involved in four of six communities within the zoonotic sharing network, highlighting their significant role.

Zoonotic pathogens like WNV and Avian Influenza viruses (AIVs) have significantly increased in incidence and geographic distribution over the past decade [7,8]. Wild birds act as primary reservoirs for both WNV and AIVs, facilitating their spread across large distances [5,9]. WNV has expanded across America and Europe with rising cases in humans [9,10], equines [11] and birds [12], highlighting its public health impact [13]. WNV ranked among the most severe zoonotic diseases in Europe in 2023 and 2024, characterized by the highest hospitalization rate among reported cases and the highest case fatality rate in 2023 [14]. Also, recent outbreaks of highly pathogenic avian influenza have significantly impacted animal and public health, affecting poultry, dairy cattle, farmed mink, and wild species [15], posing pandemic risks. As a result, avian influenza and West Nile fever have been identified by the European Commission as priority pathogens for establishing a coordinated surveillance system under the One Health approach. Importantly, a surveillance strategy that integrates both serological and virological monitoring of sentinel bird species in urban environments could facilitate the early detection of increased risks associated with pathogen transmission to humans [16].

The risk of zoonotic diseases is significantly heightened in wildlife that comes into close contact with humans or inhabits urban areas [17]. Studies have shown that increased interactions between humans and wildlife can result in higher rates of disease spillover, as seen in outbreaks of zoonotic infections such as WNV [18]. Furthermore, some of the wild bird species inhabiting urban environments are present in high densities (especially pigeons, sparrows, swallows and martins), leading to a high probability of human contact and disease spillover. For example, it has recently been shown that urban wildlife poses a growing zoonotic threat, with birds harboring high levels of potentially dangerous pathogens, sparking concerns over public health risks [19].

Passive surveillance, where information on disease events is brought to the attention of Veterinary Authorities without actively looking for it [20], is the main system for monitoring wildlife diseases in Europe [21]. Each Member State has its own routine monitoring protocols for animal health, food safety, and human diseases, but these often do not include wildlife [21]. Active surveillance provides additional value by enabling the early detection of the virus, complementing passive mandatory notifications that are primarily based on the observation of clinical signs. This information is crucial for understanding disease dynamics, assessing risks related to zoonoses, and describing the geographic distribution of pathogens [20]. Notably, active surveillance should be focused on areas with high population density, like cities and nearby regions, where contagious diseases can spread more easily [22]. In this context, by 2017, 19 out of 22 countries in the Eastern Mediterranean Region had established sentinel surveillance systems for severe acute respiratory infections (SARIs) and influenza-like illnesses (ILIs) [23,24]. Specifically, surveillance efforts are crucial in the Iberian Peninsula, a key region for avian virus dynamics at the crossroads of major flyways, but a comprehensive overview is still lacking [25].

**Figure 1:**
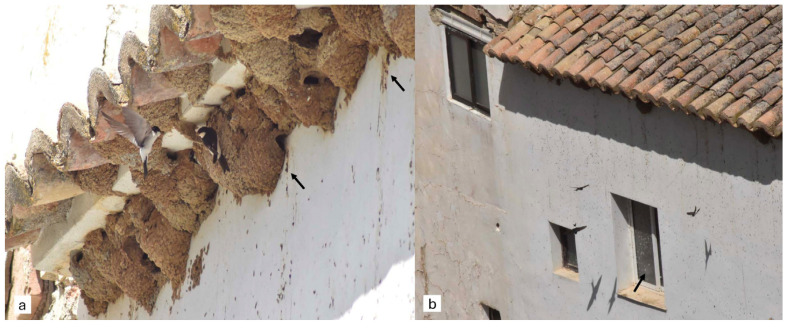
Breeding colony of the house martin located beneath the rooftop of a building in an urban environment. (**a**) Adults exhibiting nesting behaviour beneath the roof tiles. (**b**) Rooftop and exterior wall of the building supporting the colony. Black arrows represent accumulations of bird droppings.

The house martin (*Delichon urbicum*) is a migratory passerine that nests in large colonies, often in close contact with humans (Figure 1a,b). This species is typically found in urban areas and rural settings, forming breeding colonies across Europe, North Africa, and the Palearctic. During the winter months, house martins migrate to sub-Saharan Africa [26]. Birds that nest in close proximity to humans, such as house martins, may serve as indicators of the circulation of zoonotic pathogens, including viruses like WNV and AIV. Therefore, they should be included in routine surveillance programs for zoonotic diseases [27]. In this study, we analyze the presence of antibodies against flaviviruses and avian influenza and aim to detect amplicons of the matrix and nucleoprotein sequences of AIV in house martins from southern Europe. Evaluating the circulation of these zoonotic pathogens in urban environments allows us to assess the spillover risk and the potential emergence of zoonotic diseases in humans.

## 2. Material and Methods

### 2.1. Study Site and Sampling Methods

The study was carried out in a breeding colony of house martins in Badajoz (38°53′10.3″ N 6°55′32.3″ W), southwest Spain. A total of 266 birds were captured using a mist nest between June 2023 (N = 223) and July 2024 (N = 43) (Table S1). Each bird was ringed with an alphanumeric metal ring followed by age assessment based on plumage differences [28]. Birds were classified into two different groups; (i) juveniles (N = 96) (fledglings hatched in the year of sampling) and (ii) adults (N = 170) (birds that hatched in previous breeding seasons). All birds captured in 2024 were juveniles.

### 2.2. Detection of Avian Influenza Virus Infection

**Figure 2:**
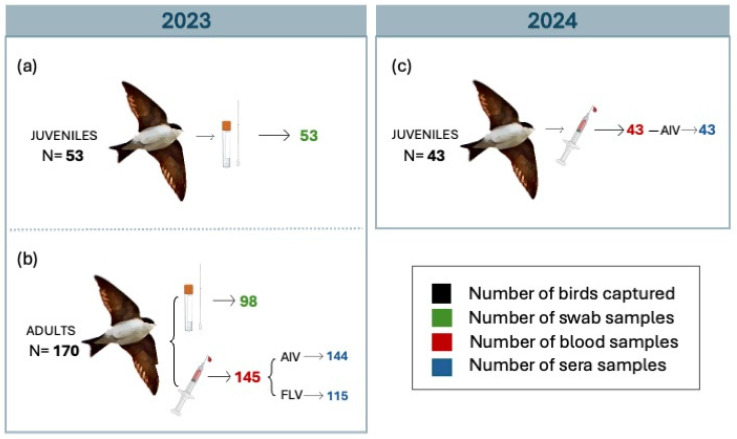
Sample size distribution by age group and year for house martins (*Delichon urbicum*). Diagram showing sample size by age group and year. (**a**) Juveniles from 2023, (**b**) Adults from 2023 and (**c**) Juveniles from 2024. The number of house martins captured each year is indicated in bold. The total sample size across all groups was 266 birds. The number of swabs collected is shown in green, the number of blood samples collected is shown in red and the number of sera samples collected is shown in blue.

In 2023, a total of 151 oropharyngeal swab samples were collected from juveniles (N = 53; Figure 2a) and adults (N = 98; Figure 2b). Swab samples were obtained by gently inserting a sterile swab (DeltaSwab Virus, Deltalab, S.L., Barcelona, Spain) into the oral cavity to reach the oropharynx, followed by a swirling motion. Shortly after, swabs were stored in tubes containing viral transport medium and stored under refrigeration at 4 °C until RNA extraction. Viral RNA was extracted from samples using a MagMax™ Pathogen RNA/DNA kit (Thermo Fisher Scientific, Waltham, MA, USA), according to the manufacturer’s instructions. To ensure purity, RNA purification was performed, followed by RNA quantification.

Extracted RNA samples were stored at −80 °C until RT-qPCR was performed using the VetMAX™-Gold AIV Detection Kit (Thermo Fisher Scientific, USA). To ensure the reliability of the results, the kit includes the Influenza Virus-Xeno™ RNA Control Mix (Thermo Fisher Scientific, USA) as a positive control for RT-qPCR components and the Xeno™ RNA Control (Thermo Fisher Scientific, USA) as an internal control to monitor RNA extraction efficiency and detect potential PCR inhibitors. The detection process was carried out using RT-qPCR, with threshold cycle (Ct) values analyzed to assess amplification efficiency and compare relative viral RNA presence in the tested samples. This kit was used as a screening tool to detect the viral matrix and nucleoprotein sequences of avian influenza. Because this kit is not designed to detect the surface glycoproteins haemagglutinin and neuraminidase, it is unable to determine the viral subtype.

Molecular analyses, including RNA extraction, purification and quantification, RT-qPCR and ELISA assays, were performed by the Bioscience Applied Techniques Service of the University of Extremadura (SAIUEx).

### 2.3. Detection of Avian Influenza and Flavivirus Antibodies

Blood samples were taken by puncture of the jugular vein with sterile insulin syringes. The volume of blood collected from each bird never exceeded 1% of its body mass. When possible, a minimum sample volume of 120 µL was used for each individual. Samples from juveniles collected in 2023 were excluded due to the insufficient volume of blood obtained. Therefore, in 2024, juveniles were selectively captured to obtain blood samples of sufficient volume. A total of 188 blood samples were collected, including adults (N = 145; Figure 2b) and juveniles (N = 43; Figure 2c) from 2024. Following manipulation, all birds were released unharmed at the capture site.

Samples were placed in sterile Eppendorf tubes at room temperature to allow coagulation and then kept in ice boxes during the sampling process. In the laboratory, coagulated blood samples were refrigerated at 4 °C. All samples were centrifuged within 24 h of collection at 11,000 rpm for 10 min to separate the serum from the cell pellet. When a sufficient volume of serum was available (at least 60 µL), the serum sample was divided into two aliquots: one duplicate for the detection of antibodies against AIV and the other for the detection of antibodies against flavivirus. The minimum volume of serum required for the detection of AIV antibodies was approximately 7 µL, whereas for flavivirus it was 50 µL. If the sample volume was insufficient to prepare two aliquots, the serum sample was preferably used for the detection of AIV antibodies. Sera samples were stored at −80 °C until further analysis.

Subsequently, 187 sera aliquots from adults sampled in 2023 (N = 144; Figure 2b) and juveniles sampled in 2024 (N = 43; Figure 2c) were tested for the presence of antibodies against AIV using a highly sensitive and specific ELISA for detection of antibodies against avian influenza in all species (CK401 AI Multi, BioCheck, Smart Veterinary Diagnostics, Reeuwijk, The Netherlands). This kit is designed to detect antibodies against the viral nucleoprotein.

**Figure 3:**
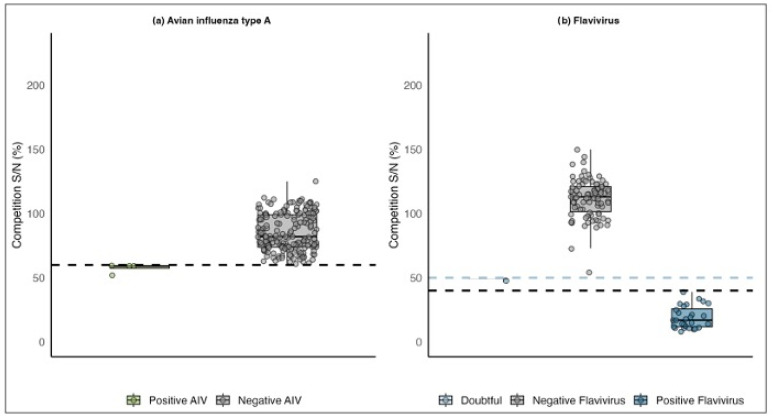
Box plot illustrating S/N ratio values for positive and negative ELISA results for (**a**) avian influenza and (**b**) flavivirus. Jittered points represent individual sample values. The dashed line in panel (**a**) represents the 60% S/N threshold for positive AIV samples (green), while in panel (**b**), the dashed lines indicate the 40% S/N threshold for positive flavivirus samples (blue) and the 50% S/N threshold for doubtful samples (light blue). The cut-off values for S/N ratios were established as recommended by the manufacturer. The median and the lower and upper quartiles are shown.

Positive samples were determined using signal (S) to noise (N) ratio and titer range. S/N ratio is defined as the proportion of the sample response divided by the negative control or background response [29]. Endpoint antibody titers were calculated following the manufacturer’s instructions using the S/N value measured at a 1:50 dilution using the following equation: ${log}_{10}(\mathrm{Titer})\;=\;-1.0\;\times \;{log}_{10}(S/N)\;+\;2.75$. The cut-off values for S/N ratios were established at 60%, as recommended by the manufacturer. Thus, to determine seropositivity to AIV, a serum sample was considered negative for AIV antibodies if the titer value was lower than 938 and/or the S/N ratio was greater than 0.60. In addition, a sample was considered positive for AIV if the titer value was greater than 938 and the S/N ratio was lower than or equal to 0.60 (Figure 3a).

Additionally, a subset of 115 aliquots of sera samples from adults (Figure 2b) was tested for the presence of antibodies against flavivirus using the ID Screen Flavivirus Competition kit (Innovative Diagnostics, Grabels, France). This Competitive ELISA kit enables the detection of a broad range of anti-pr-E flavivirus antibodies, including those against WNV, JEV, TBEV, USUV, ZIKAV, and DENV, across multiple host species. Positive flavivirus samples were determined using the S/N ratio. The cut-off values for S/N ratios were established at 40%, as recommended by the manufacturer. A serum sample was considered negative for antibodies against flavivirus if the S/N ratio was greater than or equal to 0.50. Furthermore, a sample was considered positive for antibodies against flavivirus if the S/N ratio was lower than or equal to 0.40 (Figure 3b). Sera samples with S/N ratio values between 0.40 and 0.50 were considered as doubtful (Figure 3b).

## 3. Results

The data used in this study consisted of blood and oropharyngeal swab samples collected from 266 house martins. A total of 151 oropharyngeal swab samples collected from juveniles and adults from 2023 were tested for avian influenza infection (Figure 2a,b). None of these samples were found positive for AIV (Table S2).

Additionally, a subset of 187 aliquots of sera samples from adults (Figure 2b) and juveniles from 2024 (Figure 2c) was tested for the presence of antibodies against AIV. Four of these samples were positive for AIV (Table S3; Figure 3a). The overall seroprevalence of AIV was 2.13% (95% C.I: 0.8–5.3%). All positive samples had a titer range above 938 and a S/N ratio below 60% (Table S3; Figure 3a). Remarkably, none of the 2024 samples (juveniles) were found to be positive for AIV antibodies.

Similarly, a subset of 115 aliquots of sera samples from adult house martins from 2023 was tested for the presence of antibodies against flaviviruses, since the test does not allow for differentiation between individual viruses (Figure 2b). Twenty-eight samples tested positive (Table S4; Figure 3b). The overall prevalence of infection was 24.34% (95% C.I: 17.4–32.9%). All positive samples for flavivirus exhibited a S/N ratio below 40% (Figure 3b). In addition, one sample was classified as doubtful as it had a S/N ratio of 48%. Notably, none of the birds tested by competitive ELISA were positive for both AIV and flavivirus.

## 4. Discussion

Active surveillance of wild birds living in close contact with humans is crucial for assessing the risk of zoonotic diseases [21]. This approach is particularly important in urban areas, as it enables early detection of spillover risks from zoonoses that have seen significant increases in both incidence and geographic spread over the past decade, such as WNV and AIV [20,27]. In this study, we analyzed the presence of AIV and antibodies against flaviviruses and AIV in house martins to assess the circulation of these pathogens within urban settings. Although avian influenza infection was not detected in house martins, our results revealed an overall seroprevalence exceeding 2% for avian influenza and 24% for flaviviruses in adult individuals sampled in 2023, indicating previous exposure to these viruses in this species. Our findings highlight the potential role of the house martin as a bridge host for avian influenza viruses and flaviviruses.

Synanthropic wild birds, those that thrive in close proximity to humans, can pose significant zoonotic risks, as they may serve as reservoirs for various parasites, pathogens, and diseases [5,30]. They can also act as bridge hosts, facilitating the transmission of zoonotic pathogens to other wild bird species and domestic poultry [30,31,32], probably through contamination of shared water sources and outdoor facilities [33]. Although the prevalence rates of avian influenza viruses are generally low among Passeriformes [34], their viral shedding and susceptibility to avian influenza viruses varies between species [35]. Swallows have been identified as possible bridge species that could transmit AIV from wild to domestic birds [31,34]. Moreover, the widespread presence of *Hirundinidae* species in urban settings underscores the importance of understanding their role in AIV ecology and spillover events [30]. House martins commonly build their nests on balconies or beneath roof overhangs (Figure 1a), which can increase human exposure to zoonotic viruses and pathogens through contact with potentially infected avian droppings (Figure 1b). However, the role of the house martin in AIV transmission remains less studied compared to other hirundine species [31,34,36].

Importantly, our results revealed that over 2% of adult birds showed antibodies against AIV. Although these results are based on non-subtyped ELISA, this study represents the first report of seropositive individuals for avian influenza virus in *D. urbicum* captured in Spain. Conversely, Šekler et al. [37] reported no evidence of antibodies against AIV in this species. During their southward migration to Africa, Passeriformes may contact waterfowl and share habitats, potentially exposing them to avian influenza [38]. Antibody detection in avian influenza infected birds typically occurs between 7 and 21 days post-exposure, peaking around 14 days and declining below detection levels by 12 weeks [39]. This may explain the low number of seropositive birds observed here, as sampling occurred about 20 weeks after their arrival from their African winter quarters [40]. Hence, our findings suggest that these birds have gained immunity from a previous infection, likely reflecting prior exposure to avian influenza virus in Spain. Further studies with earlier and more frequent sampling could enhance the likelihood of detecting antibodies against avian influenza in this species. Alternatively, the observed low seroprevalence may reflect limited exposure to AIV or high mortality among infected birds, with very few individuals recovering from infection [41]. Interestingly, no juvenile house martins showed antibodies against AIV, suggesting either low transmission of AIV at the Iberian breeding grounds or high mortality following primary infection in immunologically naïve individuals [42]. Nevertheless, these hypotheses should be verified by actively monitoring this house martin population and applying complementary methods, such as haemagglutination inhibition assays, molecular subtyping or pathogenicity assessment, to identify specific viral subtypes in breeding and wintering areas.

In southern Europe, the peak season for avian influenza activity typically occurs during the autumn and winter months [16,43]. We did not find any house martins infected with avian influenza virus, indicating that they were not shedding the virus at sampling in early summer. Similar findings have been reported in other PCR-based studies. For instance, Mižáková et al. [44] found only one positive out of the 13 house martins tested in Slovakia. Likewise, Lebarbenchon et al. [45] reported no AIV positives among the 10 house martins tested in southern France via RT-qPCR. These results are consistent with studies on other synanthropic passerines. For example, Ringenberg et al. [32] evaluated 266 wild synanthropic birds across eight species and found no positive cases for influenza A virus. Similarly, Le Gall-Ladevèze et al. [46] detected AI viral RNA in only nine out of 1938 wild birds tested using RT-PCR targeting AIV genes.

These findings may also be explained by the infection dynamics of AIV, which often involve rapid declines in viral loads following initial infection, as confirmed by experimental studies. Ellis et al. [39] directly inoculated European starlings (*Sturnus vulgaris*) with low-pathogenic AIV and observed that most began shedding viral RNA within 24 h post-inoculation. However, viral shedding declined rapidly, reaching near zero levels across all individuals by seven days post-inoculation. Similarly, Root et al. [35] tested three synanthropic bird species (starlings, house sparrows, and pigeons) for highly pathogenic avian influenza and found none shed detectable virus at seven days post-infection. Alternatively, the absence of infection in this study could be attributed to the high mortality rates associated with avian influenza in bird populations [47]. Supporting this hypothesis, it has been suggested that infected birds may die rapidly after infection, reducing their likelihood of being captured compared to healthy individuals. In addition, these results could be related to the sampling protocol. Oropharyngeal swabbing has been proposed as a good sampling technique that provides high detection sensitivity for AIV [48], and combining this method with cloacal swabs may have further increased virus detection [49]. These biases could lead to an underestimation of the true prevalence of the disease within the population [32,50].

Emerging vector-borne zoonotic arboviruses within the family *Flaviviridae*, such as WNV and Usutu virus (USUV), are expanding their incidence and geographic distribution across Europe, posing significant health and economic challenges [51]. Previous studies suggest that house martins may serve as a potential bridge host for flaviviruses, such as WNV [52] and USUV [53]. As long-distance migrants, they have a higher likelihood of exposure to these flaviviruses compared to non-migratory native or exotic birds [52]. In this study, we detected a flavivirus-group seroprevalence of over 24% in house martins. Since previous studies have documented the circulation of zoonotic flaviviruses like WNV in house martins within the study area [12], along with the presence of competent vectors (such as *Culex pipiens* and *Culex univittatus* subgroup) [52], our findings emphasize the importance of monitoring this bird species. House martins may serve as reservoirs for future flavivirus zoonoses and facilitate local virus circulation within avian communities.

Moreover, the flavivirus seroprevalence observed in our study (24.34%) is similar to the rates reported within the same house martin population by Marzal et al. [12] (21.55%) and Ferraguti et al. [52] (28.37%) between 2018 and 2020. These numbers suggest a widespread flavivirus circulation within this synanthropic species, potentially raising the risk of viral transmission to humans. Nevertheless, direct comparison of seroprevalence is challenging, as the commercial ELISA kits used for detecting WNV antibodies and other flaviviruses may vary in sensitivity and specificity [54,55]. For instance, the ELISA kit used by Marzal et al. [12] and Ferraguti et al. [52] is specific to WNV but shows partial cross-reactivity with other flaviviruses like USUV and Bagaza virus [56]. Although cross-reactivity with antibodies against other flaviviruses cannot be completely excluded, it is considered less likely in the present study. We used a competitive ELISA kit to detect antibodies against West Nile virus (WNV), Usutu virus (USUV), and other flaviviruses. Due to limited serum volume, virus neutralization assays could not be performed. Therefore, the detected antibodies represent an immune response to flavivirus exposure occurring either in Africa or after arrival in Europe. Future studies incorporating neutralization assays and molecular methods for viral subtyping will help clarify the identity and origin of the circulating flaviviruses and determine their seroprevalence in this species.

## 5. Conclusions

This study provides the first evidence of AIV-seropositive *Delichon urbicum* individuals captured in Spain. Our results indicate prior exposure to avian influenza viruses and to viruses within the flavivirus group in this house martin population. The observed seroprevalence suggests that viruses within these groups may be circulating in urban environments, although further virological and molecular investigations are required to confirm their identity and zoonotic potential. Recognizing synanthropic bird species like house martins as key indicators is vital for early detection and risk assessment. These insights are essential for informing One Health strategies, and implementing such integrated approaches can enhance early warning systems, guide public health interventions, and ultimately reduce the risk of future zoonoses outbreaks.

## Data Availability

All the data presented in this manuscript is available and can be obtained from the corresponding author upon reasonable request.

## Supplementary Materials

The following supporting information can be downloaded at https://www.mdpi.com/article/10.3390/ani16060913/s1, Table S1: Overview of data from 266 house martins tested for avian influenza virus and flavivirus; Table S2: Results of RT-qPCR targeting the viral matrix and nucleoprotein genes of avian influenza virus; Table S3: ELISA results, including S/N ratio and antibody titer values for avian influenza virus; Table S4: ELISA results, including S/N ratio for flaviviruses.
